# Supplementary material for: Associations between Florida counties’ COVID-19 case and death rates and meaningful use among Medicaid providers: Cross-sectional ecologic study
Source: PLOS Digit Health. 2022 Jun 2;1(6):e0000047. doi: 10.1371/journal.pdig.0000047 (PMC9931361; doi:10.1371/journal.pdig.0000047)
Supplement: S1 Text — (DOCX) [file pdig.0000047.s002.docx]

| COVID DEATHS LSMEANS FOR f1542513 % Persons 65+ in Deep Poverty 2013-17 |
| --- |

The Mixed Procedure

| **Model Information** | |
| --- | --- |
| **Data Set** | WORK.ALL |
| **Dependent Variable** | COVDEATHSPOPPCT1K112020 |
| **Covariance Structure** | Diagonal |
| **Estimation Method** | REML |
| **Residual Variance Method** | Profile |
| **Fixed Effects SE Method** | Model-Based |
| **Degrees of Freedom Method** | Residual |

| **Class Level Information** | | |
| --- | --- | --- |
| **Class** | **Levels** | **Values** |
| **RECM_USE** | 2 | NO YES |
| **VOLCALC** | 2 | 0 1 |
| **PTDDS** | 2 | NO YES |
| **Df0453810** | 2 | <MEDIAN >=MEDIAN |
| **DF0453910** | 2 | <MEDIAN >=MEDIAN |
| **DF0454210** | 2 | <MEDIAN >=MEDIAN |
| **DF1193515** | 2 | <MEDIAN >=MEDIAN |
| **DF1193615** | 2 | <MEDIAN >=MEDIAN |
| **DF0978117** | 2 | <MEDIAN >=MEDIAN |
| **DF1322617** | 2 | <MEDIAN >=MEDIAN |
| **DF1332117** | 2 | <MEDIAN >=MEDIAN |
| **Df1549817** | 2 | <MEDIAN >=MEDIAN |
| **Df1553417** | 2 | <MEDIAN >=MEDIAN |
| **Df0679518** | 2 | <MEDIAN >=MEDIAN |
| **DF0978117** | 2 | <MEDIAN >=MEDIAN |
| **DF1542513** | 2 | <MEDIAN >=MEDIAN |

| **Dimensions** | |
| --- | --- |
| **Covariance Parameters** | 1 |
| **Columns in X** | 31 |
| **Columns in Z** | 0 |
| **Subjects** | 1 |
| **Max Obs per Subject** | 8437 |

| **Number of Observations** | |
| --- | --- |
| **Number of Observations Read** | 8748 |
| **Number of Observations Used** | 8437 |
| **Number of Observations Not Used** | 311 |

| **Covariance Parameter Estimates** | |
| --- | --- |
| **Cov Parm** | **Estimate** |
| **Residual** | 0.01067 |

| **Fit Statistics** | |
| --- | --- |
| **-2 Res Log Likelihood** | -14222.5 |
| **AIC (Smaller is Better)** | -14220.5 |
| **AICC (Smaller is Better)** | -14220.5 |
| **BIC (Smaller is Better)** | -14213.5 |

| **Type 3 Tests of Fixed Effects** | | | | |
| --- | --- | --- | --- | --- |
| **Effect** | **Num DF** | **Den DF** | **F Value** | **Pr > F** |
| **RECM_USE** | 1 | 8421 | 0.19 | 0.6645 |
| **VOLCALC** | 1 | 8421 | 164.35 | <.0001 |
| **PTDDS** | 1 | 8421 | 0.09 | 0.7664 |
| **Df0453810** | 1 | 8421 | 453.45 | <.0001 |
| **DF0453910** | 1 | 8421 | 122.03 | <.0001 |
| **DF0454210** | 1 | 8421 | 462.31 | <.0001 |
| **DF1193515** | 1 | 8421 | 81.48 | <.0001 |
| **DF1193615** | 1 | 8421 | 27.08 | <.0001 |
| **DF0978117** | 1 | 8421 | 3022.83 | <.0001 |
| **DF1322617** | 1 | 8421 | 1595.94 | <.0001 |
| **DF1332117** | 1 | 8421 | 38.34 | <.0001 |
| **Df1549817** | 1 | 8421 | 507.36 | <.0001 |
| **Df1553417** | 1 | 8421 | 644.17 | <.0001 |
| **Df0679518** | 1 | 8421 | 3799.55 | <.0001 |
| **DF1542513** | 1 | 8421 | 183.89 | <.0001 |

| **Least Squares Means** | | | | | | |
| --- | --- | --- | --- | --- | --- | --- |
| **Effect** | **% Persons 65+ in Deep Poverty 2013-17** | **Estimate** | **Standard Error** | **DF** | **t Value** | **Pr > \|t\|** |
| **DF1542513** | **<MEDIAN** | 0.8349 | 0.004469 | 8421 | 186.82 | <.0001 |
| **DF1542513** | **>=MEDIAN** | 0.7845 | 0.003365 | 8421 | 233.16 | <.0001 |

| **Differences of Least Squares Means** | | | | | | | | | |
| --- | --- | --- | --- | --- | --- | --- | --- | --- | --- |
| **Effect** | **% Persons 65+ in Deep Poverty 2013-17** | **% Persons 65+ in Deep Poverty 2013-17** | **Estimate** | **Standard Error** | **DF** | **t Value** | **Pr > \|t\|** | **Adjustment** | **Adj P** |
| **DF1542513** | **<MEDIAN** | **>=MEDIAN** | 0.05047 | 0.003722 | 8421 | 13.56 | <.0001 | SMM | <.0001 |
