# Supplementary material for: Associations between Florida counties’ COVID-19 case and death rates and meaningful use among Medicaid providers: Cross-sectional ecologic study
Source: PLOS Digit Health. 2022 Jun 2;1(6):e0000047. doi: 10.1371/journal.pdig.0000047 (PMC9931361; doi:10.1371/journal.pdig.0000047)
Supplement: Attachment — Submitted filename: PLOSDHResponse to reviewer040522.docx [file pdig.0000047.s003.docx]

**Response to Reviewers**

**Reviewer #1:**

Comments to the Authors: This is overall a well-written manuscript presenting the "Associations between Florida Counties’ COVID-19 Case and Death Rates and Meaningful Use Among Medicaid Providers: Cross-Sectional Ecologic Study".

- It clearly states the problem to be solved and specified the nature of the knowledge sought in the study.
- Manuscript is well organized and flows well. The language is unbiased and the length is appropriate.
- The writing give substantive evidence of the authors’ familiarity with the subject, literature and concepts presented in the study. References are scholarly, current and appropriate
- The content is innovative and builds and advances body of knowledge of Ecologic Studies / Public Health Data and Health Informatics regarding to the associations between Florida Counties’ COVID-19 Case and Death Rates and Meaningful Use Among Medicaid Providers:
- The design of the research is sound. The description of the methodology, findings and relevancy is identifiable, precise and accurate.
- The Data analysis is clear, relevant and thorough to support the conclusion.

**RESPONSE:** We thank Reviewer 1 for these comments and taking the time to review our paper.

**Reviewer #2:**

*Critiques*

1. The authors analyse an interesting and novel aspect of the COVID-19 pandemic, and they took a lot of variables into consideration. An extensive statistical analysis is also provided. There is one thing which might worth mentioning: the incident rates are based on the reported case numbers, but not all COVID-19 cases are detected. People with no or mild symptoms might not report in the health care system, and limitation of testing kits or human work force might result in underestimating the case numbers. Do you have any information regarding these issues in the analysed regions, especially if there are any differences among the provides which might affect your conclusions?

**RESPONSE:** Thank you for your careful read and consideration of our findings. We acknowledge that likely there were cases with no or mild symptoms that were not reported to the state via a PCP. Also, there were no COVID-testing sites at that time; subsequently testing sites were established to provide free COVID-19 testing to those with and without symptoms. Also, home testing kits became available in more recent months. Since this time, several publications report lower than expected case rates of COVID-19 in Florida. We have included several references in the limitation section.

2. The manuscript contains some minor editing errors, including some typos on page 5 and an "[Error! Bookmark not defined.]" on page 12

**RESPONSE:** As advised, the typo on page 5 has been revised including throughout the paper to reflect Meaningful Use. The "[Error! Bookmark not defined.]" on page 12 has been updated to reflect reference #31.

**Reviewer #3:**

*Critiques*

1. The authors present a study of COVID-19 death, case and case-fatality rates (CFR) across Florida counties. They report that death rates and CFR, but not case rates, were associated with Meaningful Use of EHRs achieved by Medicaid providers. The authors claim that their findings have implications for policymaking, giving evidence that government support to adopt EHRs among Medicaid providers has beneficial effects.

**RESPONSE:** We thank Reviewer 3 for these comments and taking the time to review our paper thoroughly. We have revised the verbiage to further clarify the alignment with HealthyPeople 2030 Health IT objectives to increase the proportion of office-based physicians that have the necessary information available electronically at point of care. Additionally, this revision further supports the goals of The Agency for Health Care Administration (AHCA) toward achieving the desired technology in Florida, to increase value for providers and encourage patient-centered care through improved access to relevant and meaningful information at point-of-care. These revisions are now included in the author summary and the conclusion sections of the paper.

**Major points:**

1. While the authors report significant differences, the effect sizes are very small and differences are well within standard deviations. This raises the suspicion that the small p-values are simply due to the large sample size, and not a true underlying difference. The authors should confirm that the reported p-values are correct and should comment on this issue.

**RESPONSE:** Thank you for your careful read and consideration**.** Reported p-values are correct. Analysis of the data was performed using available data from reliable sources (Florida Department of Health which is updated daily, US Census Bureau and Area Health Resources Files), all open-access government generated files, and all of which support content validity. We acknowledge that the effect size is small, and with larger sample sizes, power increases. However, we are analyzing all data from a population of Florida’s Medicaid providers and COVID-19 rates, so a power analysis was not planned. Effect sizes have to be considered within the context of the prevalence of an attribute in a population, distribution of the exposure variable, and severity of the outcome; thus in this study, a small effect size is acceptable. To put our findings into perspective, there were over 80 million people enrolled in Medicaid by 2021; assuming that 50% of providers for this U.S. population achieved Meaningful Use, as per Florida, then applying our estimate of .1% to 40 million results in 40,000 lives that may have been saved if electronic health records advanced use was implemented. Thus, we see a noteworthy disparity that should be investigated further as to specifics of how EHRs improve safety, effectiveness, patient-centeredness, communication, education, timeliness, efficiency, and equity. [https://www.healthit.gov/faq/what-are-advantages-electronic-health-records].

2. In particular, given the small effect sizes, it does not seem justified to derive from this a strong case for policies regarding EHR adoption, especially since the authors could not confirm that patients of Medicaid providers who achieved Meaningful Use were of similar risk to patients of providers who did not. The authors need to at least provide additional justification for why they believe their data provides good evidence for extending government support beyond 2021.

**RESPONSE:** We thank Reviewer 3 for this comment. We acknowledge the limitations in available data, and have lessened the emphasis on policy making by framing it in the context of existing goals of both HealthyPeople 2030 Health IT and The Agency for Health Care Administration. We agree that additional justification is needed to further explain extending government policies already in place, and have updated the manuscript to reference our support of the alignment with Healthy People 2030 Health IT objectives and the goals of The Agency for Health Care Administration (AHCA).

3. **A**. The authors performed a large number of comparisons and, as is to be expected, found some significant associations. However, in addition to small effect sizes (point 1), any real causative associations would also need to be plausible. For example, it is difficult to imagine a plausible mechanism by which EHR adoption by dentists could be causally related to COVID-19 case rates.

**RESPONSE:** We present all results of our analyses, including those for dentists in Tables 1-3. In conjunction with Table 3, we reemphasize that the interactions between practice of dentistry and Meaningful Use (Table 3) are secondary analyses. We state that only 55 dentists achieved Meaningful Use, representing only 1.5% of those achieving Meaningful Use. In the Limitations section, we discuss potential unreliability of these findings given the small sample size of dentists achieving Meaningful Use.

**We have omitted the following sentences in the Results section (deemphasis) because of potential unreliability, although these results are reported in Table 3 for transparency:**

*“Among dentists, COVID-19 case rates were significantly lower if they achieved Meaningful Use (P<.001), compared with other providers who may or may not have achieved Meaningful Use.”*

*“In these secondary analyses, the estimated reported COVID-19 case rates associated with dentists who did not achieve Meaningful Use were significantly greater than those for dentists who achieved Meaningful Use, relative to this comparison for providers with other specialties. These findings suggest that interventions directed toward Medicaid dentists to increase Meaningful Use could result in notable improvement in mitigation strategies.”*

**We have added the following sentence in the Results section to read as follows:**

*“Additionally, there were only 55 dentists of the 3723 Medicaid providers who achieved Meaningful Use (1.5%), and thus reliability of findings may be of concern.”*

B. Why would COVID-19 death rates be lower in counties with a higher concentration of persons ages 65 years and older living in deep poverty, but at the same time positively correlate with higher rates of those living in poverty? The authors need to provide their rationale behind not excluding such associations as unsound. Did the authors conduct further analyses to gain insight about this finding?

**RESPONSE:** We have attached the results of the multivariate analyses supporting this finding. Interpretation of the result that older adults not living in poverty is associated with increased death rates requires understanding of where the deaths occurred. For example, if deaths were concentrated in long-term care facilities in which residents had to have a minimal amount of money to secure residence, and the concentration of residents in these facilities was greater than alternative homes, and then the result is plausible. Analyses involved many variables and comparisons, many of which cannot be readily interpreted without further qualitative information from providers and residents. Authors have not as yet undertaken these ancillary studies.

4. Even if there is a real difference in COVID-19 death rates and CFR among Medicaid providers who did and did not achieve Meaningful Use, the authors do not provide evidence that EHR adoption was the causal factor. It could be that providers who achieved Meaningful Use offer better care quite independent of EHR adoption. Again, they would need to give additional justifications for deriving policy implications from their results.

**RESPONSE:** First, as stated in the text, “Although we label the design as cross-sectional and report associations, we note that our study focuses on Medicaid providers enrolled in HITECH between 2012 and 2016 with a minimum of two years of follow-up occurring prior to the pandemic, and thus the design has a longitudinal component.” We are careful to emphasize the study assesses associations and not causality. Second, regarding policy implications, please see our response to Reviewer 3, Critique 1.

**Minor points:**

1. The authors correctly state that Meaningful Use was captured before the COVID-19 pandemic, and therefore case and death rates are not confounded by it. However, their Methods suggest that the last follow-up took place in 2018. It would strengthen their study if they could include more recent data showing that those providers who sustained Meaningful Use in 2018 were still doing so during the COVID-19 pandemic.

**RESPONSE:** Extending the period of followup may have resulted in some providers being followed and other not; this could introduce length time bias. If we wanted to extend the study through 2018, we would have needed data through 2020 which we did not have.

2. A reference is broken on p.12.

**RESPONSE:** As advised, The "[Error! Bookmark not defined.]" on page 12 has been updated to reflect reference #31.

**Reviewer #4:**

This work addresses the impact and differences between Medicaid providers who did and did not implement electronic health records and achieve Meaningful Use as it relates to Florida county-level cumulative COVID-19 death, case and case fatality rates, taking into consideration the county level demographics, socio-economic and clinical markers, and healthcare environment.

This work had several findings. Below are some of the findings:

(a)	The findings from this work show that there were significant differences in the cumulative incidence rates of COVID-19 deaths and case fatality rates between the 5025 Medicaid providers not achieving Meaningful Use and the 3723 that achieved Meaningful Use.

(b)	County level characteristics such as lower median household income, greater concentrations of persons of African American or Black race, higher unemployment, and higher concentrations of those living in poverty and without health insurance, were independently associated with increased COVID1-9 death rates and case fatality rates.

(c)	The association between Florida counties public health outcomes and Meaningful Use achievement most likely had less to do with using electronic health records for reporting of clinical outcomes and had more to do with using electronic health records for coordination of care

Critiques

My comments

I enjoyed reading this paper. It was well written and the analysis and results were well reported and I thought the discussions section was detailed and addressed the analysis, results, and findings from this work.

I think this paper should be accepted. It is my opinion that the research community will benefit from this paper being published. Also, this work and its findings could potentially encourage other researchers to conduct similar work in various states across the United States.

Below is a minor comment In this work, one of the county-level characteristics associated with lower COVID-19 death rates was a higher concentration of person’s ages 65 and older living in deep poverty. Did the authors conduct further analyses to gain insight about this finding?

**RESPONSE:** Thank you for your comments and review. Please see the response in Section 3. B above. This was also noted in the paper Monestime et al. using the same data but without COVID-19 outcomes (2021).
